# Notes and News

**Published:** 1903-06-27

**Authors:** 


### NOTES AND NEWS.

THE Pathological Society of London will hold its annual meeting at Oxford on July 4th.

THE new buildings of the serum department of the Jenner Institute will be opened at Elstree on July 3rd.

THE Earl of Derby, President of the Brompton Consumption Hospital has given £1,000 to the country branch of the hospital.

THE Asylum Works Association have presented a tablet to Colney Hatch Asylum, in testimony of the bravery of the attendants and nurses on the occasion of the memorable fire.

DR. FINSEN has suggested that a commission should be appointed to inquire into the question of the general use of the red light in small-pox hospitals and in all cases of small-pox.

THERE seems to be a general slight recrudescence of the plague in most of the previously infected districts, and in India the death-rate from the disease during the same month last year was only half as large as this year.

THIS year the Enno Sander Prize of the United States Association of Military Surgeons has been awarded to a British military surgeon, Major F. Smith, D.S.O., of the Royal Army Medical Corps. The award was received with expressions of much generous pleasure on the part of the American Association. The subject of the essay was the early diagnosis of typhoid fever.

WE are glad to see that Sir John Gorst is upholding the view we expressed some time ago that the much-lauded physical exercises for poor school children were a mistake unless it was ascertained that the children were properly nourished, so as to stand the strain. The Edinburgh Commission has revealed what a small percentage of school children are physically fit, and, as Sir John Gorst pointed out in the House of Commons, a similar investigation would probably reveal a like condition in our Metropolis.

A GRAND massed band concert will be held in aid of the Union Jack Club at the Crystal Palace on Saturday, July 4th, at 3.30. The New Zealand Band and ten other bands will take part.

A NEW dietary scale, allowing four meals a day to men in the Navy, is the outcome of the recent inquiry into the question of naval diet. The new dietary includes a greater variety also.

MR. WILLIAM SINCLAIR, M.B.C.M., has been appointed medical superintendent of the Aberdeen Royal Infirmary, in place of Dr. Angus, who has accepted the post of superintendent of the Aberdeen and District Lunatic Asylum.

RIGOROUS and careful exercise of the regulations of the Food Adulteration Act has reduced the percentage of adulteration of milk in Manchester to 3½, whilst in the Metropolis it amounts to about 15 per cent., and some districts equal 34 per cent.

THE outcome of the third meeting of medical officers of Life Insurance companies held in Paris seems to have been a general concensus of experience and opinion in favour of admitting doubtful lives at increased premiums to the benefits of life insurance.

THE Alien Emigration Question has been taken up by the Medical Officer of Health for the Port of London, and the Port Sanitary Authorities are prepared to carry out any measures that may be thought desirable in respect to the overcrowding on emigrant ships arriving at the Port.

A GREAT deal of public interest was excited last week in radium, and the possibilities opened up by the discovery, by demonstrations and addresses at the Royal Institute and at the Royal Society. Already its applicability to the relief of all forms of disease is being tested. Dr. London, of the Imperial Russian Institute of Experimental Medicine, is endeavouring to utilise the light to help the blind to read and write.

THE social functions in connection with the annual meetings of the British Medical Association form no small part of the programme. This year the enticements are greater than ever for medical men if not for their wives. The Swansea Bay Golf Club is throwing open its doors to the Association and an international match is, if possible, to be arranged. The neighbourhood offers other sporting attractions which will be made the most of on the occasion.

THE Semi-Teetotal Pledge Association, in announcing the success of their "no drinks between meals" campaign, profess to be surprised that teetotalers are enrolling themselves under their banner, but that they refuse the license to drink at all which the new pledge allows. There is not quite so much surprise outside the organisation, for few have regarded the movement as anything but a widening of the aims of the teetotal movement, or as practically the thin edge of the wedge! Otherwise there could be little justification for glorifying mere common sense, which is all that "no drinks between meals" means.

